# Grape seed proanthocyanidins improves growth performance, antioxidative capacity, and intestinal microbiota in growing pigs

**DOI:** 10.3389/fmicb.2024.1501211

**Published:** 2024-11-20

**Authors:** Yuyang Zheng, Yan Li, Bing Yu, Zhiqing Huang, Yuheng Luo, Ping Zheng, Xiangbing Mao, Jie Yu, Huize Tan, Junqiu Luo, Hui Yan, Jun He

**Affiliations:** ^1^Institute of Animal Nutrition, Sichuan Agricultural University, Chengdu, Sichuan, China; ^2^Key Laboratory of Animal Disease-Resistant Nutrition, Chengdu, Sichuan, China; ^3^Wens Foodstuff Group Co., Ltd., Yunfu, China

**Keywords:** GSP, growth performance, nutrient digestibility, antioxidant capacity, intestinal microbiota, growing pigs

## Abstract

Grape seed proanthocyanidin (GSP) is a kind of plant polyphenols with a wide variety of biological activities. In this study, we explored the effect of dietary GSP supplementation on growth performance, nutrient digestibility, and intestinal microbiota in growing pigs. A total of 180 growing pigs (30.37 ± 0.31 kg) were randomly assigned to five treatment groups, each consisting of six replicate pens with six pigs per pen. The pigs received either a basal diet (control) or a basal diet supplemented with GSP at 15, 30, 60, or 120 mg/kg. The trial lasted for 33 days, and blood and fecal samples were collected for biochemical measurements. GSP supplementation at a dose from 30 to 120 mg/kg decreased the ratio of feed intake to gain (*F*:*G*) (*p* < 0.05). GSP also increased the digestibility of dry matter, crude protein, ether extract, and gross energy (*p* < 0.05). GSP supplementation at 30 mg/kg increased the serum concentrations of immunoglobulin (Ig) A (*p* < 0.05). Interestingly, GSP supplementation at 60 mg/kg decreased the serum concentrations of urea and malondialdehyde (*p* < 0.05). However, the serum concentrations of glutathione peroxidase and total superoxide dismutase were significantly increased upon GSP supplementation (*p* < 0.05). Importantly, GSP supplementation at 120 mg/kg significantly increased the abundance of the phylum Firmicutes, but decreased the abundance of phylum Bacteroidetes and Epsilonbacteraeota in the feces (*p* < 0.05). Moreover, GSP supplementation significantly elevated the abundance of genus Lactobacillus, but decreased the abundance of genus Prevotellaceae NK3B31 (*p* < 0.05). Dietary GSP supplementation improves the growth performance and nutrient digestibility in growing pigs, which may be associated with enhancement of the antioxidative capacity, as well as improvement in gut microbiota. This study may promote the use of GSP in animal nutrition and the feed industry.

## 1 Introduction

Oxidative stress is characterized by excessive production of reactive oxygen species (ROS) within the cell, which leads to disruption of the redox balance in the body (Bhor et al., 2004). Various factors contribute to oxidative stress in pigs, including weaning stress, mycotoxin contamination, social interactions, and the feeding environment (Novais et al., 2021; Zhao et al., 2013; Yang et al., 2020). Accumulating evidence showed that the modulation of the diet of pigs can also significantly reduce oxidative stress. For instance, dietary supplementation of probiotics during the weaning period was found to attenuate oxidative stress and improve the growth performance of piglets by improving the gut microbiota and inhibiting intestinal inflammation (Xiang et al., 2020). Moreover, dietary supplementation of an antioxidant compound can enhance feed conversion and increase oxidative defense in finishing pigs (Zhu et al., 2022).

Proanthocyanidin is a natural pigment that is abundant in numerous plant species and belongs to a kind of flavonoid compound. Grape seed proanthocyanidin (GSP), is extracted from grape seeds, which is a polymeric compound formed by the condensation of phenolic compounds with trihydroxyflavones. Their chemical structure comprises the core anthocyanin structure, wherein a benzene ring is attached to a tricyclic flavonoid scaffold, featuring hydroxyl and methoxy functional groups (Rodríguez et al., 2019). Prior research has demonstrated that GSP possesses a range of bioactive properties. For instance, GSP exhibits strong antioxidant properties, capable of scavenging free radicals and mitigating oxidative stress, thereby aiding in cellular protection against oxidative damage (Chedea et al., 2010). It also has anti-bacterial (Guo et al., 2020), anti-inflammatory (Tyagi et al., 2013), anti-viral (Ignea et al., 2013), immunomodulatory (Tong et al., 2011), etc. Recently, GSP has been utilized by a wide variety of animal species. For instance, GSP can improve the health status of tilapia and enhance its growth rate (Zhai et al., 2016). GSP has also been reported to enhance rumen fermentation function in cattle, thereby increasing nutrient digestibility (Ma et al., 2023). Moreover, GSP can protect the integrity of the goose intestinal barrier, increase the abundance and diversity of cecal microflora, and promote the growth of beneficial bacteria (Deng et al., 2023).

Although a number of studies revealed a prominent health-promoting effect of GSP, clear evidence establishing direct links among GSP dosage, product origin, developmental stage, and animal species is lacking. Furthermore, the biological events influenced by GSP are still not fully understood in terms of their underlying mechanisms. This study investigated the impact of dietary GSP supplementation on growth performance and intestinal microbiota in growing pigs. We found that GSP significantly improved growth performance in growing pigs, which may be associated with improvement in nutrient digestibility, antioxidative capacity, and intestinal microbiota. This study not only sheds light on the mechanisms underlying GSP’s effects on growth in pigs, but may also facilitate its application in animal nutrition and the feed industry.

## 2 Materials and methods

### 2.1 Ethics statement

The research underwent submission and approval by the Committee on Animal Care Advisory of Sichuan Agricultural University, with the authorization number SICAU-2021-007. The experiment procedures were conducted in accordance with the Guidelines for the Care and Use of Laboratory Animals.

### 2.2 Experimental design and diet

A total of 180 pigs (Duroc × Landrace × Yorkshire) with an initial BW of 30.37 ± 0.66 kg (90 barrows and 90 gilts) were allocated to five treatments based on Randomized Complete Block Design. Each treatment group comprised six replicate pens (6 pigs per pen). For each treatment, three replicate pens were utilized to house the barrows and another three pens were utilized to house the gilts (pigs of the same gender were housed in a pen). The treatments were a control diet formulated to meet the requirements from NRC (2012) nutrient requirements (Table 1) with no addition of GSP, and the control diet with four increasing levels of GSP (15, 30, 60, and 120 mg/kg). The GSP was supplied by Fengpeng Biotechnology Co., Ltd. (Guilin, China) and contains 86.81% proanthocyanidin oligomers, 1.52% catechin, 2.41% epicatechin, and 0.98% proanthocyanidin B_2_, with a total proanthocyanidin purity of 96.58%. The feeders were refilled three times a day, and drinking water *ad libitum* throughout the experimental period (33 days). The feed intake was recorded every day. The final body weight, average daily feed intake (ADFI), average daily gain (ADG), and the ratio of feed intake to gain (*F*:*G*) were measured at the end of the experiment.

**Table 1:** Composition and nutrient levels of the experimental diets (air-dry basis, %).

Ingredient	%	Nutrient composition^3^	Contents
Corn	72.58	Analyzed nutrient levels	
Soybean meal	17.93	Gross energy, MJ/kg	18.95
Bran	2.50	Crude protein, %	18.48
Fish meal	2.50	Ether extract, %	3.12
Soybean oil	2.00	Calcium, %	0.71
Limestone	0.69	Total phosphorus, %	0.47
Dicalcium phosphate	0.58	Calculated nutrient levels	
Salt	0.30	Digestible energy, MJ/kg	14.23
l-Lysine HCl	0.31	Crude protein, %	15.88
dl-Methionine	0.03	Ether extract, %	5.08
l-Threonine	0.07	Calcium, %	0.66
l-Tryptophan	0.01	Total phosphorus, %	0.51
Choline chloride	0.15	Available phosphorus, %	0.31
Vitaminic premix^1^	0.05	SID^4^ Lysine, %	0.98
Mineral premix^2^	0.30	SID Methionine + Cystine, %	0.50
		SID Threonine, %	0.59
		SID Tryptophan, %	0.17

1 Provided the following (per kilogram of complete diet): 15,000 IU of vitamin A; 5,000 IU of vitamin D_3_; 40 IU of vitamin E; 5 mg of vitamin K; 5 mg of vitamin B_1_; 12.5 mg of vitamin B_2_; 6 mg of vitamin B_6_; 0.06 mg of vitamin B_12_; 50 mg of nicotinic acid; 25 mg of pantothenic acid; 2.5 mg of folic acid; 0.25 mg of biotin.

2 Provided the following (per kilogram of complete diet): 60.0 mg of Fe (as ferrous sulfate); 4 mg of Cu (as copper sulfate); 60.0 mg of Zn (as zinc sulfate); 2.0 mg of Mn (as manganese sulfate); 0.14 mg of I (as KI); 0.2 mg of Se (as Na_2_SeO_3_).

3 The calculated nutrient levels of the diet were obtained from the China Feed Database (2020).

4 SID = standardized ileal digestibility.

### 2.3 Sample collection

At the outset of the experiment, feed samples were gathered and preserved for subsequent analysis. During days 25–28 of the experiment, fecal samples were collected daily for four consecutive days from each replicate to assess nutrient digestibility. Hydrochloric acid at a concentration of 10% was added to the fecal samples for nitrogen fixation, and then the samples were dried in an oven at 60°C. The dried samples were then pulverized in a high-speed pulverizer and passed through 0.45 pm filters for chemical assay. Additionally, a portion of fecal samples was collected and stored at −80°C for 16S rDNA sequencing. Approximately 15 mL of blood was drawn from the anterior vena cava of one randomly selected pig from each of the six pens per group on day 33 of the experiment. The blood samples were then centrifuged at 3,000 × *g* for 20 min at 4°C to isolate the serum.

### 2.4 Apparent total tract nutrient digestibility analysis

Dried and ground feed and fecal samples were utilized for nutrient digestibility analysis, employing acid-insoluble ash (AIA) as an endogenous indicator. The AIA in diet and fecal samples was measured as described by National National Standard (2009). The diet and fecal samples were evaluated for dry matter (DM), crude protein (CP) (Method 976.05; AOAC, 2007), and ether extract (EE) (Method 922.06; AOAC, 2007). An automated oxygen bomb calorimeter (Model 6,400, Parr, United States) was used to evaluate the gross energy (GE). The apparent total tract nutrient digestibility for all parameters was calculated using the following formula:


$$\left[\left(1-A1F2/A2F1\right)\times 100\right],$$

in which A1: is the AIA content of the diet, A2: is the AIA content of fecal, F1: is the nutrient content of the diet, F2: is the nutrient content of fecal. The diet was analyzed for calcium and total phosphorus content by inductively coupled plasma spectroscopy (method 985.01 A, B, and C; AOAC, 2007). The calculated nutrient levels of the diet were obtained from the China Feed Database (2020).

### 2.5 Serum biochemical, immunoglobulin, and antioxidant parameters

Serum biochemical parameters were measured using an Olympus automatic analyzer (Shanghai, China). Utilizing ELISA kits (MEIMIAN, Yancheng, Jiangsu, China), the concentrations of immunoglobulins (Ig) A (MM-090502), G (MM-040302), and M (MM-040202) were measured. Commercial kits from Nanjing Jiancheng Bioengineering Institute (Nanjing, Jiangsu, China) were utilized to measure the concentrations of total antioxidative capacity (T-AOC) (Cat. No. A015-1-2), glutathione peroxidase (GSH-Px) (Cat. No. A005-1-2), catalase (CAT) (Cat. No. A007-1-1), total superoxide dismutase (T-SOD) (Cat. No. A001-1-1), and malondialdehyde (MDA) (Cat. No. A003-1-2).

### 2.6 Diversity and composition of the bacterial community

From 0.5 g of fecal samples, nucleic acids were extracted with the Stool DNA kit (TIANGEN, China) in accordance with the manufacturer’s guidelines. The analysis of bacterial community diversity and composition was conducted using the Quantitative Insights Into Microbial Ecology (QIIME) software package. The PCR amplification focused on the V4 region of the 16S rRNA gene, employing primers 515F (5’-GTGYCAGCMGCCGCGGTAA-3′) and 806R (5’-GGACTACHVGGGTWTCTAAT-3′). The PCR conditions were as follows: initial denaturation at 94°C for 1 min, followed by 1 cycle; denaturation at 94°C for 20 s, annealing at 54°C for 30 s, and extension at 72°C for 30 s for 25–30 cycles; final extension at 72°C for 5 min; held at 4°C. Amplification was carried out using a Verity Thermocycler (Applied Biosystems). PCR products were purified through electrophoresis on a 2% agarose gel. Desired bands were excised from qualified samples and purified using the Zymoclean Gel Recovery Kit (D4008), quantified with a Qubit 2.0 Fluorometer (Thermo Scientific), and mixed in equimolar amounts. Sequencing was conducted using the PE250 method with the Illumina NovaSeq 6,000 SP Reagent Kit V1.5.

Paired-end reads were merged using FLASH. Sequences were demultiplexed based on barcodes with SABRE, followed by barcode trimming, and quality control was performed using QIIME2. Within QIIME2, sequences were denoised and chimeras were removed using the Deblur algorithm, generating an ASV feature table and feature sequences. A 97% similarity threshold was used to define operational taxonomic units (OTUs). The analysis of community composition was performed in R, employing various data transformations and utilizing the ggplot2 package for visualization. The alpha diversity was analyzed in R, with PD index calculations performed using the Picante package, while other indices were calculated using the Vegan package. Bray–Curtis distance was computed using the vegdits function in Vegan, and PCoA analysis was conducted with the ape package.

### 2.7 Sample size calculation and statistical analysis

Data were analyzed by IBM SPSS 27.0 software (Statistical Product and Service Solutions, Inc., United States). In the context of growth performance and nutrient digestibility, each pen is regarded as the experimental unit; however, for other experimental data, individual pigs are treated as the experimental unit. The growth performance data were analyzed utilizing Linear Mixed Model (LMM), considering dietary treatment and sex as fixed effects with an interaction term for dietary treatment × sex, while considering pen as a random effect. Other data were analyzed using a general linear model (GLM), with dietary treatment and sex as fixed effects with an interaction term for dietary treatment × sex. The effects of dietary treatment were evaluated using one-way ANOVA, followed by Duncan’s multiple comparison test to assess mean differences. Results are expressed as mean ± standard error of the mean (SEM), with statistical significance set at *p* < 0.05 and trends defined as 0.05 < *p* < 0.10.

## 3 Results

### 3.1 Growth performance and nutrient digestibility

As shown in Table 2, dietary GSP supplementation at a dose from 30 to 120 mg/kg significantly increased the ADG and decreased the *F*:*G* ratio of the pigs (*p* < 0.05). Moreover, with dietary GSP supplementation, the digestibility of dry matter, crude protein, ether extract, and gross energy increased quadratically (quadratic, *p* < 0.05).

**Table 2:** Effect of GSP on growth performance and nutrient digestibility in growing pigs.

Items	GSP (mg/kg)					SEM	*p*-value		
	0	15	30	60	120		LMM	Linear	Quadratic
Initial weight, kg	30.39	30.29	30.42	30.33	30.42	0.66	0.75	0.98	1.00
Final weight, kg	57.99	58.88	59.99	59.00	59.53	0.41	0.11	0.28	0.41
ADFI, kg/d	1.95	1.95	1.93	1.89	1.96	0.02	0.30	0.73	0.58
ADG, kg/d	0.84^c^	0.87^b^	0.90^a^	0.87^ab^	0.88^ab^	0.01	<0.01	0.23	0.30
*F*:*G*	2.35^a^	2.26^ab^	2.16^b^	2.19^b^	2.24^b^	0.03	<0.01	0.23	0.18
DM, %	85.66^b^	86.64^ab^	87.52^a^	87.84^a^	86.93^a^	0.26	0.01	0.04	0.01
CP, %	82.63^c^	83.07^bc^	84.59^ab^	85.11^a^	84.45^ab^	0.33	0.02	0.01	0.02
EE, %	66.77^b^	66.84^b^	69.39^a^	70.03^a^	69.73^a^	0.40	<0.01	<0.01	<0.01
GE, %	88.34^c^	89.03^bc^	89.48^b^	90.68^a^	89.35^bc^	0.24	<0.01	0.03	0.02

Data are shown as mean ± SEM (*n* = 6 replicates per treatment).

^a,b,c^Means lacking a common uppercase superscript differ (*p* < 0.05) using Duncan test.

GSP, Grape Seed Proanthocyanidin; SEM, standard error of the mean; LMM, Linear Mixed Model; ADFI, average daily feed intake; ADG, average daily gain; *F*:*G*, feed to gain ratio; DM, dry matter; CP, crude protein; EE, ether extract; GE, gross energy.

### 3.2 Serum biochemical parameters

As shown in Table 3, dietary GSP supplementation did not affect serum biochemical parameters such as the ALT, AST, CK, and TP (*p* > 0.05). Nevertheless, the serum concentration of urea decreased quadratically by GSP supplementation (quadratic, *p* < 0.05).

**Table 3:** Effects of dietary GSP on serum biochemical parameters in growing pigs.

Items	GSP (mg/kg)					SEM	*P*-value		
	0	15	30	60	120		GLM	Linear	Quadratic
ALT, U/L	52.29	48.02	45.42	44.54	45.29	1.99	0.77	0.22	0.37
AST, U/L	64.83	39.63	34.00	33.43	37.95	4.59	0.24	0.06	0.04
ALP, U/L	103.83	105.00	104.17	112.00	104.83	5.60	0.99	0.83	0.96
ALB, g/L	27.08	24.69	23.78	25.10	28.87	0.79	0.34	0.48	0.06
TP, g/L	52.70	48.96	50.66	54.25	59.02	1.50	0.36	0.09	0.07
GLU, mmol/L	4.59	4.18	4.28	4.22	4.91	0.11	0.23	0.38	0.05
TG, mmol/L	0.59	0.58	0.57	0.54	0.70	0.05	0.88	0.59	0.63
TC, mmol/L	2.13	1.92	1.96	2.06	2.15	0.05	0.53	0.62	0.29
CK, U/L	64.83	39.63	34.00	33.43	37.95	4.56	0.24	0.06	0.04
CREA, μmol/L	85.51	72.49	83.64	80.12	84.39	2.75	0.64	0.79	0.68
UREA, mmol/L	3.15^a^	2.46^bc^	2.14^c^	2.95^ab^	3.20^a^	0.11	<0.01	0.48	< 0.01
LDH, U/L	832.30	492.27	413.02	360.13	457.98	58.36	0.12	0.03	0.01

Data are shown as mean ± SEM (*n* = 6 replicates per treatment).

^a,b,c^Means lacking a common uppercase superscript differ (*P* < 0.05) using Duncan test.

GSP, Grape Seed Proanthocyanidin; SEM, standard error of the mean; GLM, General Linear Model; ALT, alanine aminotransferase; AST, aspartate aminotransferase; ALP, alkaline phosphatase; ALB, albumin; TP, total Protein; GLU, glucose; TG, triglyceride; TC, total cholesterol; CK, creatine kinase; CREA, creatinine; UREA, urea; LDH, lactate Dehydrogenase.

### 3.3 Serum immunoglobulins and antioxidative capacity

As shown in Table 4, GSP supplementation at 30 mg/kg significantly elevated serum IgA levels (*p* < 0.05). Additionally, serum MDA levels decreased quadratically with GSP supplementation (quadratic, *p* < 0.05), while serum levels of GSH-Px and T-SOD exhibited a quadratic increase (quadratic, *p* < 0.05).

**Table 4:** Effects of GSP on serum immunoglobulin and antioxidant parameters in growing pigs.

Items	GSP (mg/kg)					SEM	*P*-value		
	0	15	30	60	120		GLM	Linear	Quadratic
IgA, μg/mL	36.39^b^	35.31^b^	42.17^a^	39.07^ab^	37.28^ab^	0.76	0.05	0.31	0.12
IgG, μg/mL	525.91	507.05	608.03	574.92	569.47	22.42	0.67	0.34	0.54
IgM, μg/mL	7.69	7.97	8.69	10.04	8.19	0.47	0.47	0.36	0.44
MDA, nmol/mL	4.13^a^	3.60^ab^	3.42^ab^	3.07^b^	3.15^b^	0.13	0.10	<0.01	0.01
T-AOC, U/mL	3.81	3.81	3.94	4.55	4.64	0.18	0.49	0.07	0.17
CAT, U/mL	2.04	2.05	2.53	2.69	2.42	0.11	0.16	0.06	0.11
GSH-Px, U/mL	526.57^c^	560.36^bc^	605.64^ab^	642.64^a^	598.11^ab^	11.48	< 0.01	<0.01	<0.01
T-SOD, U/mL	141.12^b^	147.14^ab^	149.34^ab^	157.24^a^	161.58^a^	2.34	0.04	<0.01	<0.01

Data are shown as mean ± SEM (*n* = 6 replicates per treatment).

^a,b,c^Means lacking a common uppercase superscript differ (*P* < 0.05) using Duncan test.

GSP, Grape Seed Proanthocyanidin; SEM, standard error of the mean; GLM, General Linear Model; IgA, immunoglobulin A; IgG, immunoglobulin G; IgM, immunoglobulin M; MDA, malondialdehyde; T-AOC, total antioxidant capacity; CAT, catalase; GSH-Px, glutathione peroxidase; T-SOD, total supemxide dismutase.

### 3.4 Intestinal microbiota

**Figure 1:**
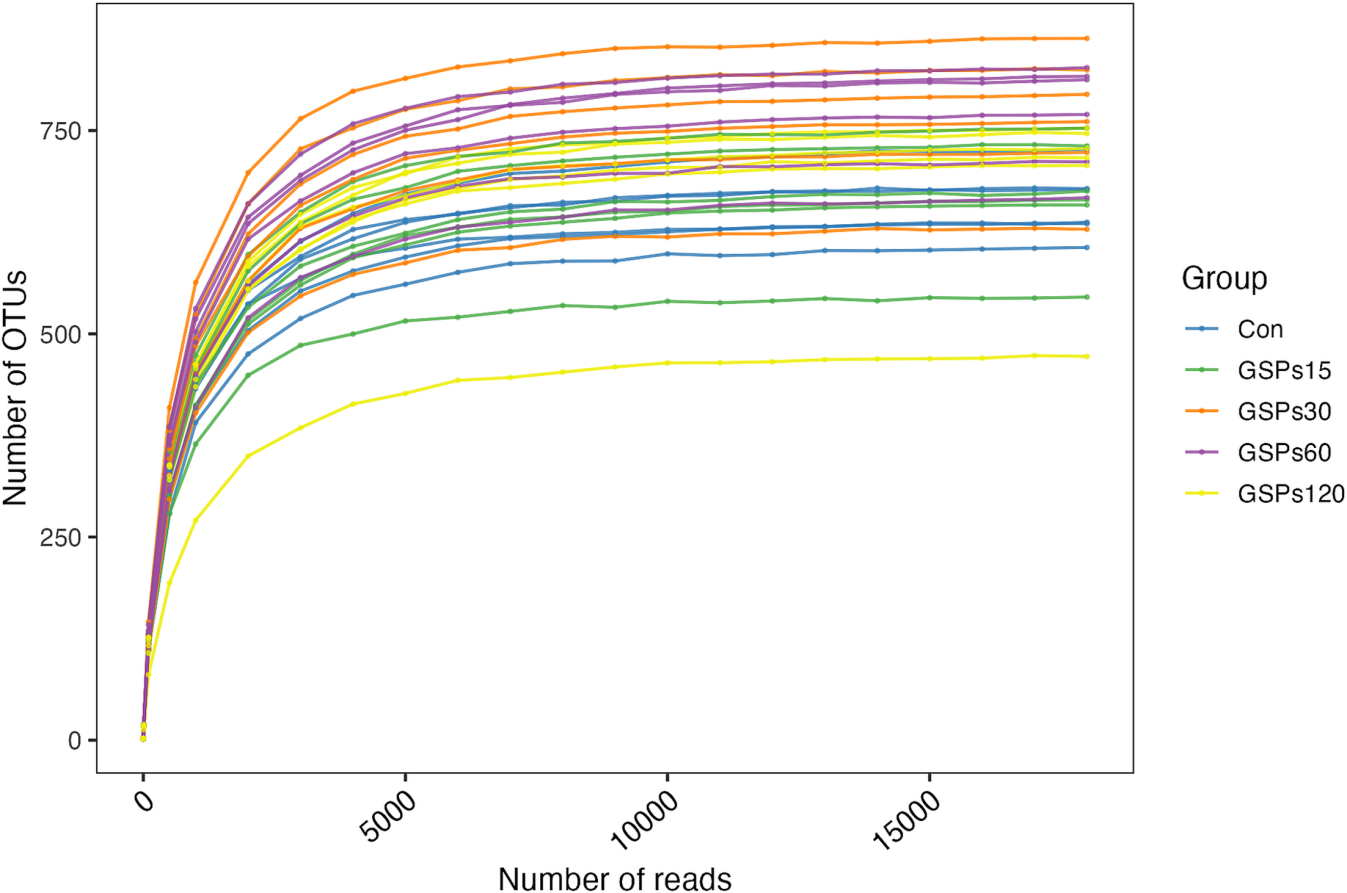
Rarefaction curves. Con, control group; GSPs15, 15 mg/kg GSP group; GSPs30, 30 mg/kg GSP group; GSPs60, 60 mg/kg GSP group; GSPs120, 120 mg/kg GSP group (*n* = 6).

Following OTU assignment and chimera checking, a total of 1,002,573 effective tags representing the 16S rRNA gene V4 region were selected from 1,027,009 tags, yielding an average of 33,419 sequences per fecal sample. The percentage of combined sequences for each sample ranged from 97.17 to 98.04% (Supplementary Table S1). The rarefaction curves indicated that the depth of sampling was adequate to assess the bacterial communities (Figure 1).

**Figure 2:**
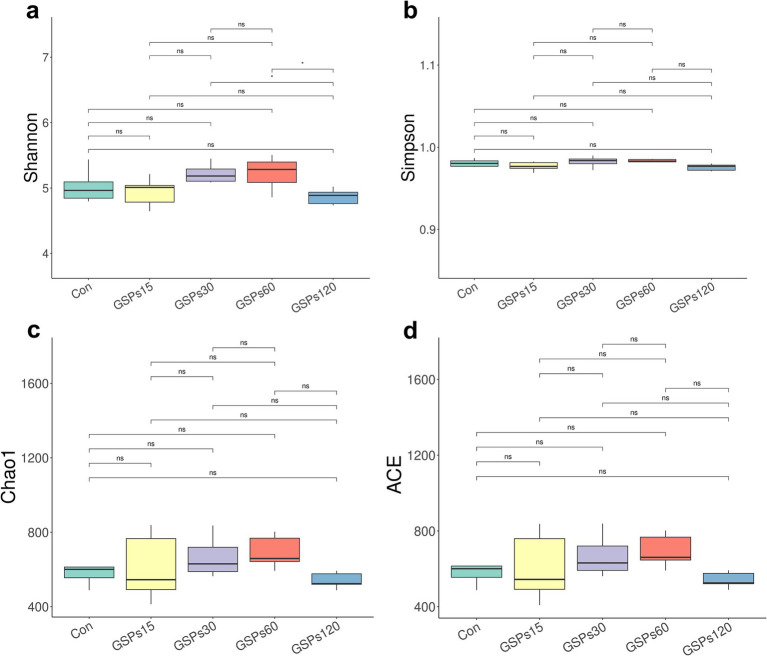
The boxplot of differences on bacterial community diversity and richness. (a) Shannon index. (b) Simpson index. (c) Chao1 index. (d) ACE index. Con, control group; GSPs15, 15 mg/kg GSP group; GSPs30, 30 mg/kg GSP group; GSPs60, 60 mg/kg GSP group; GSPs120, 120 mg/kg GSP group (*n* = 6). *Means significant difference (*p* < 0.05) between groups.

**Figure 3:**
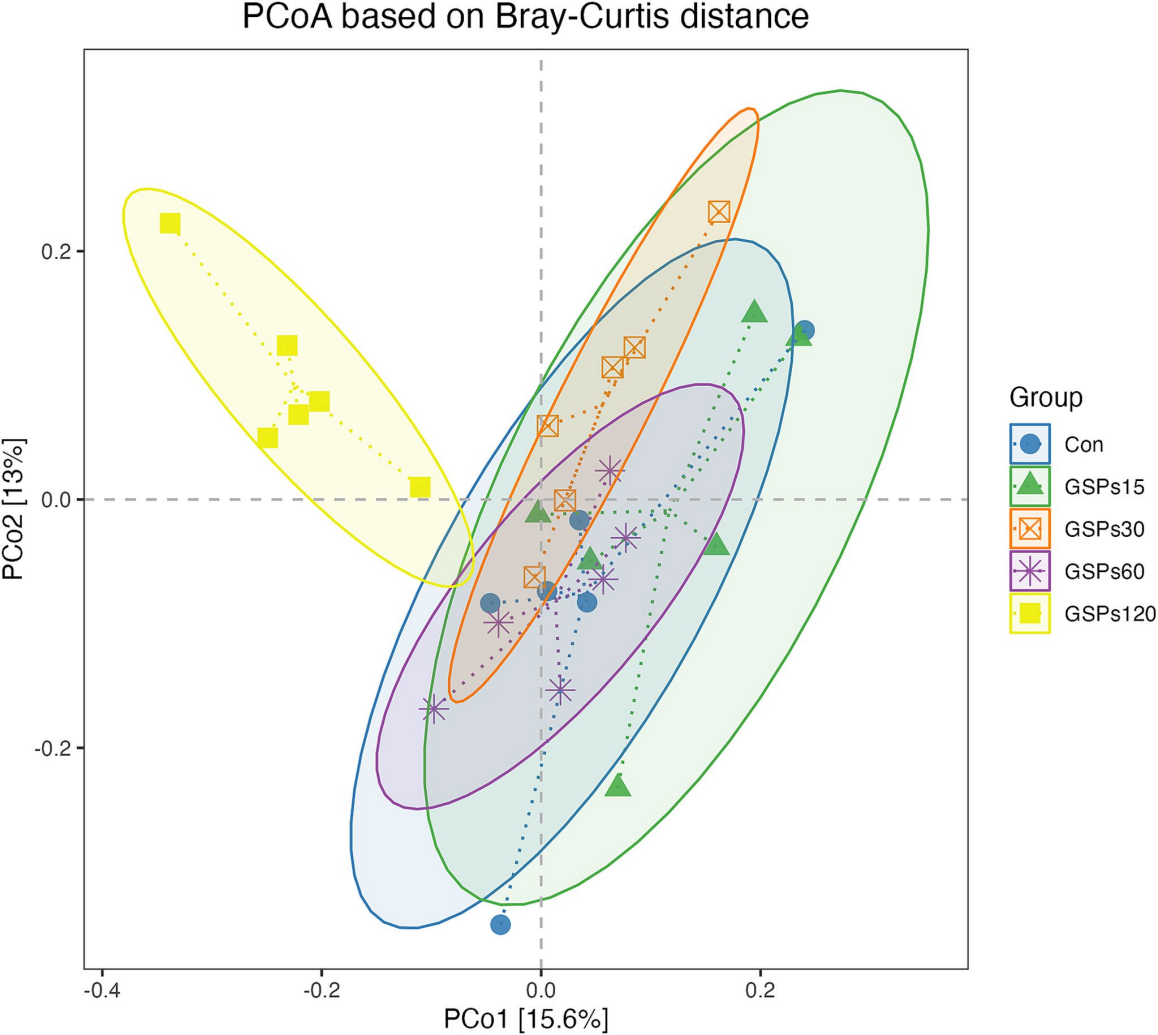
The PCoA analysis based on Bray–Curtis distance. Con, control group; GSPs15, 15 mg/kg GSP group; GSPs30, 30 mg/kg GSP group; GSPs60, 60 mg/kg GSP group; GSPs120, 120 mg/kg GSP group (*n* = 6).

The alpha diversity index is shown in Figure 2 and Supplementary Table S2. Dietary GSP supplementation has no significant differences in the alpha diversity index. Principal coordinate analysis (PCoA) based on Bray–Curtis distance metrics (Figure 3) indicated a distinct separation in microbiota composition between the 120 mg/kg GSP group and other treatment groups (*p* < 0.05).

**Figure 4:**
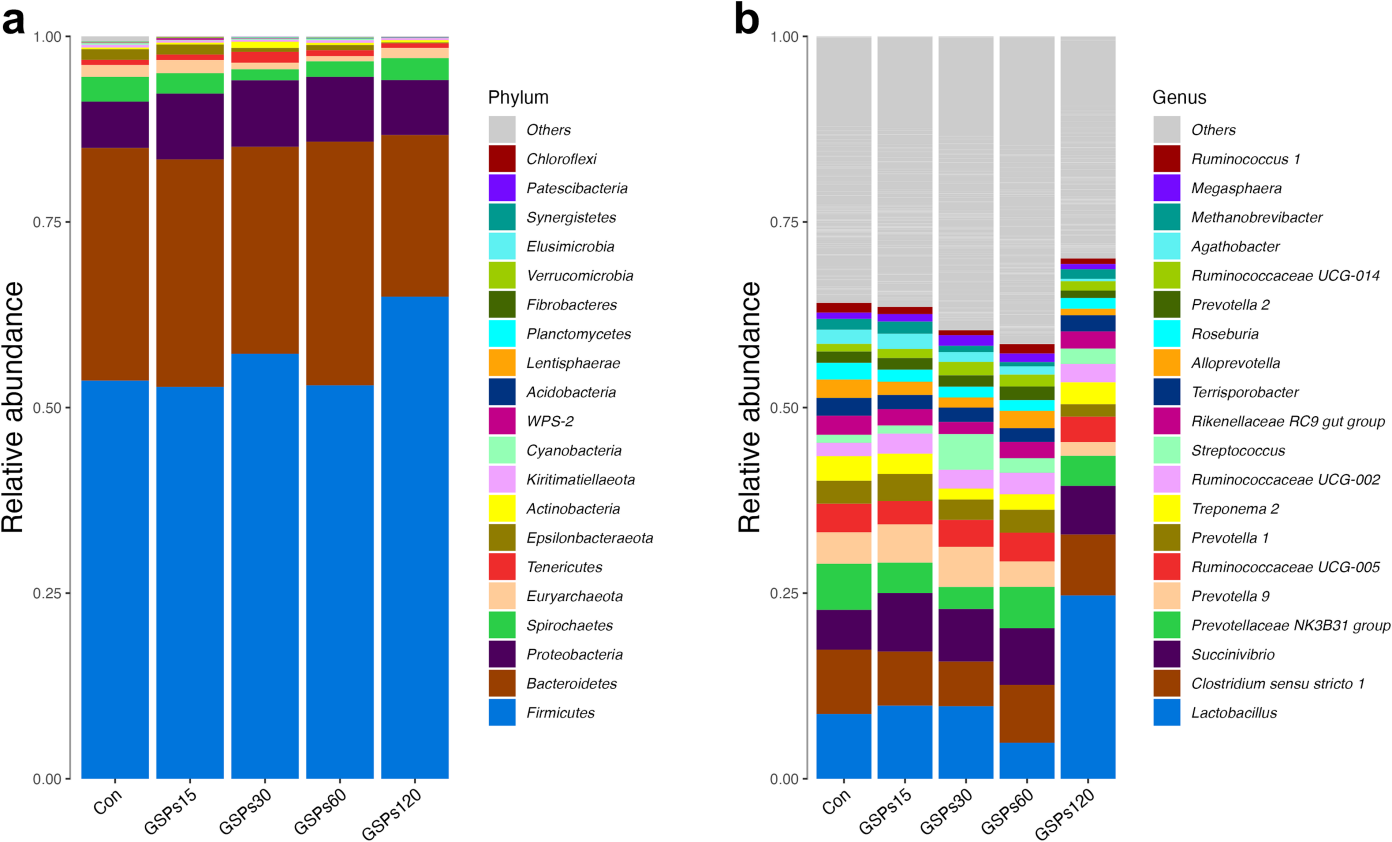
Bar graph shows the phylum (a) and genus (b) level composition of bacteria. Color coded bar plot shows the relative abundance of bacterial phyla and genus across the different samples. Con, control group; GSPs15, 15 mg/kg GSP group; GSPs30, 30 mg/kg GSP group; GSPs60, 60 mg/kg GSP group; GSPs120, 120 mg/kg GSP group (*n* = 6).

**Figure 5:**
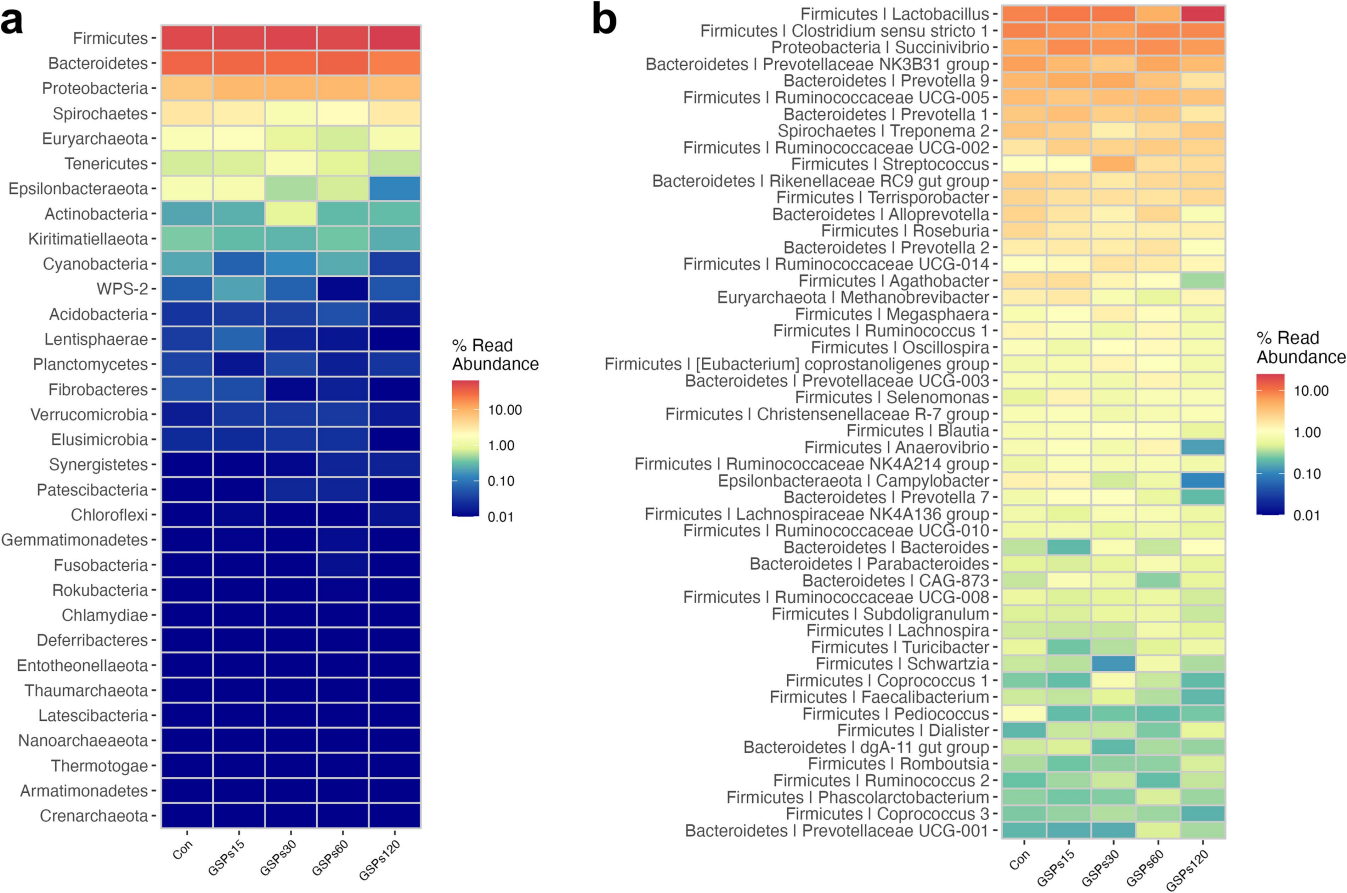
Heatmap distribution of OTUs at phylum (a) and genus (b) level. OTUs were arranged in rows and are clustered on the vertical axis. Samples are arranged vertically and are on the horizontal axis. Different colors indicate the relative abundance of taxons. Con, control group; GSPs15, 15 mg/kg GSP group; GSPs30, 30 mg/kg GSP group; GSPs60, 60 mg/kg GSP group; GSPs120, 120 mg/kg GSP group (*n* = 6).

At the phylum level, all of the qualified sequences were assigned to 21 known phyla (Figure 4A and Supplementary Table S3). Among these predominant phyla, GSP supplementation at 120 mg/kg enhanced the abundance of Firmicutes compared to the CON group (*p* < 0.05), but decreased the abundance of the phylum Bacteroidetes in fecal samples (*p* < 0.05). At the genus level, the sequences derived from fecal samples were to 21 known genera (Figure 4B and Supplementary Table S4). GSP supplementation at 120 mg/kg increased the abundance of the *Lactobacillus* (*p* < 0.05). Moreover, GSP supplementation at 120 mg/kg decreased the *Prevotellaceae NK3B31 group* and *Alloprevotella* abundance at the genus level (*p* < 0.05). The heatmap displays the abundance of selected phyla and genera across samples, clearly highlighting significant differences in distribution between the treatment groups (Figure 5).

**Figure 6:**
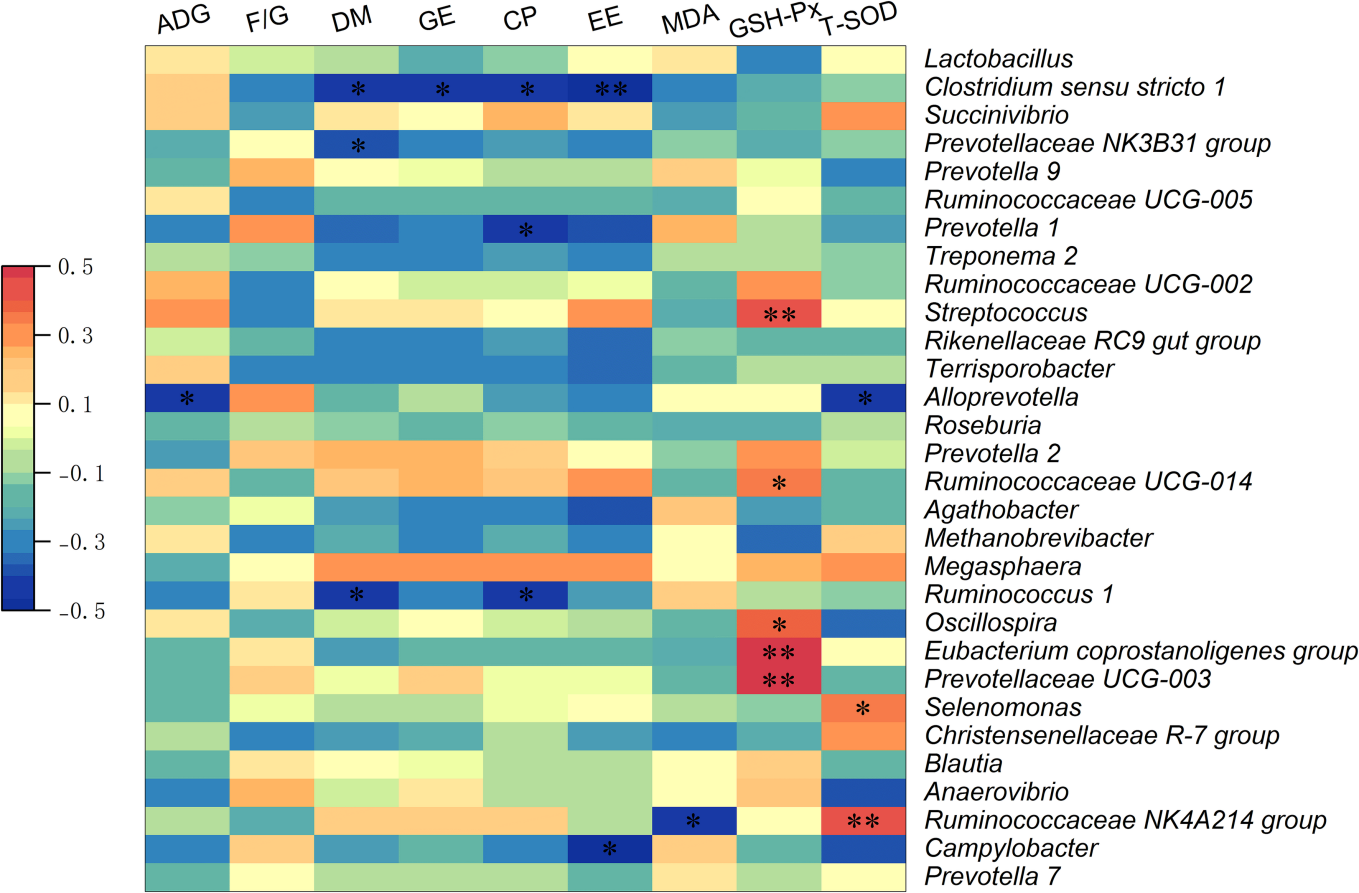
The Spearman correlation analysis of fecal microbial composition with growth performance, nutrient digestibility, and antioxidant capacity in growing pigs. Spearman correlation coefficients of ADG, *F*/*G*, nutrient digestibility (DM, GE, CP, and EE), serum MDA concentration, serum GSH-Px concentration, and serum T-SOD concentration with fecal microbiota at genus level, are displayed. The heatmap with red indicated a positive correlation, while blue represented a negative correlation. ADG, average daily gain; *F*/*G*, feed to gain ratio; DM, dry matter; GE, gross energy; CP, crude protein; EE, ether extract. *p < 0.05, **p < 0.01.

Analysis using Spearman correlation (Figure 6) demonstrated significant links between the top 30 genera of intestinal microbiota and essential metrics such as growth performance, nutrient digestibility, and antioxidant capacity in growing pigs. A notable negative correlation was observed between average ADG and the relative abundance of *Alloprevotella* (*p* < 0.05). Additionally, dry matter digestibility showed an inverse relationship with the relative abundances of *Clostridium sensu stricto 1*, the *Prevotellaceae NK3B31 group*, and *Ruminococcus 1* (*p* < 0.05). Moreover, the digestibility of GE was negatively associated with the relative abundance of *Clostridium sensu stricto 1* (*p* < 0.05). A negative correlation was found between the digestibility of CP and the relative abundances of *Clostridium sensu stricto 1*, P*revotella 1*, and *Ruminococcus 1* (*p* < 0.05). Negative associations were observed between the digestibility of EE and the relative abundances of *Clostridium sensu stricto 1* and *Campylobacter* (*p* < 0.05). Furthermore, serum MDA levels were negatively correlated with the *Ruminococcaceae NK4A214 group* (*p* < 0.05). A positive correlation was identified between serum GSH-Px levels and the relative abundances of *Streptococcus*, *Ruminococcaceae UCG-014*, *Oscillospira*, *Eubacterium coprostanoligenes group*, and *Prevotellaceae UCG-003* (*p* < 0.05). Moreover, the serum T-SOD concentration was negatively correlated with the relative abundance of *Alloprevotella*, but was positively correlated with the relative abundances of *Selenomonas* and *Ruminococcaceae NK4A214 group* (*p* < 0.05).

## 4 Discussion

In the present study, we explored the effect of dietary GSP supplementation on growth performance and nutrient digestibility in growing pigs. We found that GSP supplementation significantly increased the feed efficiency, as indicated by the decrease in the ratio of *F*:*G* and the increase in the digestibility of DM, CP, EE, and GE. This finding aligns with earlier research conducted on piglets and growing pigs (Fiesel et al., 2014; Park et al., 2014). Improved feed efficiency may be linked to enhanced immunity and antioxidative capacity resulting from GSP supplementation (Chedea et al., 2010; Hao et al., 2021; Tong et al., 2011). In this study, serum concentrations of IgA were significantly elevated by dietary supplementation of GSP at 30 mg/kg, which predominantly exists on mucosal surfaces, such as those of the respiratory and digestive tracts, where it serves as a crucial first line of defense by preventing pathogen adhesion and penetration of the mucosa (Pabst, 2012). Previous study also indicated that polyphenolic compounds, such as magnolol, can enhance amino acid absorption and protein synthesis in growing pigs, thereby maintaining immune homeostasis (Liu et al., 2023).

Oxidative stress can induce acute inflammatory response in the intestine, leading to structural and functional disruptions in the intestinal epithelium and increasing intestinal permeability (Assimakopoulos et al., 2004; John et al., 2011). Moreover, oxidative stress can also lead to oxidative modifications of digestive enzymes, resulting in decreased enzyme activity, which impairs the digestion and absorption of nutrients (Bhor et al., 2004). In recent years, various antioxidants have been utilized to enhance the antioxidant capacity of pigs. For instance, certain plant extracts such as resveratrol, *β*-glucans, daidzein, etc., have been reported to scavenge excessive free radicals in the body, effectively alleviate intestinal oxidative damage, promote villus growth and increase intestinal wall thickness, thereby enhancing feed efficiency (Goh et al., 2023; Li et al., 2021; Meng et al., 2018). The GSP has potent antioxidant properties, as the multiple hydroxyl groups in its polyphenolic structure can effectively neutralize free radicals and reduce their activity through resonance stabilization (Spranger et al., 2008). By activating the Nrf2 signaling pathway and enhancing the expression of downstream antioxidant enzymes, GSP can also mitigate oxidative stress (Xu et al., 2019). In this study, the antioxidative capacity of the pigs was significantly enhanced by GSP supplementation, as indicated by the decrease in serum concentration of MDA. The MDA is one of the end products of lipid peroxidation and can indirectly reflect the extent of oxidative damage in the body (Marnett, 1999). Moreover, GSP supplementation also enhanced the concentrations of T-SOD and GSH-Px. GSH-Px is a selenium-containing enzyme that catalyzes the reduction of hydrogen peroxide and organic peroxides to water and the corresponding alcohol using reduced glutathione as a substrate, and the T-SOD is a type of metal-containing enzyme that is the first line of defense in the detoxification of oxidative stress products (Ceballos-Picot et al., 1996; Fattman et al., 2003). These findings align with earlier reports indicating that dietary GSP supplementation can enhance serum antioxidant indices in finishing pigs through the activation of the Nrf2 signaling pathway (Feng et al., 2023).

The intestinal microbiota, which comprises all microorganisms residing in the digestive tract, is crucial for the normal functioning of digestive, immune, metabolic, and nervous systems through its balance and diversity (Álvarez et al., 2021). The microbial communities in different sections of the digestive tract exhibit significant differences in composition, whereas fecal microbiota provides a comprehensive reflection of the entire gut microbiome (Eckburg et al., 2005). Earlier research indicated that GSP significantly impacts the modulation of intestinal microbiota dysbiosis (Jin et al., 2018; Seo et al., 2017; Tao et al., 2019). In this study, PCoA analysis revealed distinct microbial community structures and compositions in the group receiving 120 mg/kg GSP compared to the other groups. This result aligns with earlier studies conducted on both mice and finishing pigs (Choy et al., 2014; Sheng et al., 2020). This change may reflect competitive displacement among microbial populations under GSP treatment, resulting in a new microbial equilibrium, which could affect nutrient absorption, immune response, and overall gut function (Biagi et al., 2016; Hertz et al., 2020).

Changes in the fecal microbiota have been shown in a previous study to be closely associated with intestinal health (Hopkins, 2001). In the present study, the phyla Firmicutes and Bacteroidetes were found to be the most predominant phyla in the fecal samples, which is consistent with a previous study in pigs (Verschuren et al., 2019). The bacteria of phylum Firmicutes can efficiently degrade plant fibers and other complex carbohydrates, producing short-chain fatty acid (Sun et al., 2022). In this study, we found that supplementation with GSP at 120 mg/kg significantly enhanced the abundance of the phylum Firmicutes, deepening our understanding of how dietary GSP can improve the bioavailability of nutrients. In animals, a major phylum present in the intestine is Bacteroidetes, which is involved in food digestion, nutrient absorption, and immune system regulation, but its overgrowth can lead to immune system imbalance (Ferolla et al., 2014; Pittayanon et al., 2019). In this study, we observed that GSP supplementation at 120 mg/kg reduced the ratio of the phylum Bacteroidetes in feces. This finding is consistent with earlier research indicating that plant polyphenols can inhibit Bacteroidetes growth, thereby affecting the host’s energy metabolism in the intestine (Xue et al., 2016). Moreover, Firmicutes and Bacteroidetes are crucial members of the gut microbiota, and they may engage in resource competition (Ley et al., 2006). The substantial increase in members of Firmicutes such as *Lactobacillus* may occupy more ecological niches and nutrient resources, thereby restricting the growth and proliferation of Bacteroidetes (Chen M. L. et al., 2016; Chen S. et al., 2016; Wang et al., 2024).

The *Lactobacillus* genus is a group of Gram-positive bacteria that can regulate the intestinal environment by producing beneficial metabolic products such as lactic acid, maintaining the acid–base balance, and inhibiting the growth of harmful bacterial populations (Dempsey and Corr, 2022). In this study, fecal samples from pigs receiving a diet supplemented with 120 mg/kg GSP showed a higher abundance of *Lactobacillus* compared to the CON group. This finding aligns with results from a previous study on another polyphenolic compound, resveratrol, which was shown to increase the levels of *Lactobacillus* in the gut, ultimately contributing to the regulation of bile acid metabolism (Chen M. L. et al., 2016; Chen S. et al., 2016). The *Prevotellaceae* is one of the major bacterial families in the large intestine of pigs, due to its ability to promote the production of short-chain fatty acids, is suggested to play an important role in intestinal metabolism (Amat et al., 2020; Zhang et al., 2021). Nevertheless, certain members of the *Prevotellaceae* family have been found to potentially exert negative effects on health by promoting inflammatory responses and contributing to disease progression. For instance, the mucin-degrading activity of the *Prevotellaceae NK3B31 group* may compromise intestinal epithelial integrity, fostering endotoxemia, inflammation, and insulin resistance (Hasain et al., 2021). Furthermore, previous research indicated that *Prevotella copri* (a member of *Alloprevotella*) exacerbates DSS-induced colitis in mice by downregulating ATF4 expression and altering the composition of intestinal microbiota (Wang and Cao, 2024). In the present study, GSP supplementation at 120 mg/kg decrease the genera abundances of the *Prevotellaceae NK3B31 group* and *Alloprevotella*. A previous study found that pigs with a low feed conversion ratio had higher abundances of taxa from the *Prevotellaceae* family in the ileum compared to those with a high feed conversion ratio (Quan et al., 2018). Consistently, the Spearman correlation analysis showed that the digestibility of DM was negatively correlated with the genus abundance of the *Prevotellaceae NK3B31 group*. Additionally, the abundance of *Alloprevotella* was found to be negatively correlated with both ADG and T-SOD concentration. Consequently, the observed dynamic changes in the intestinal microbiota community may partially account for the improvements in growth performance and nutrient digestibility noted in this study. Further studies, including fecal microbiota transplantation, are necessary to clarify the precise role and potential mechanisms underlying the biological events modulated by the intestinal microbiota.

## 5 Conclusion

In summary, dietary GSP supplementation not only increases the growth performance, but also improves the nutrient digestibility of growing pigs. The improved nutrient digestibility by GSP is likely linked to the enhancements in antioxidative capacity and improvement in the intestinal microbiota. Based on our findings, dietary supplementation of 30 and 60 mg/kg GSP may be optimal for growing pigs. This study may enhance the application of GSP in animal nutrition and the feed industry.

## Data Availability

The raw sequence data for the 16S rRNA presented in this study can be found in online repositories. The names of the repository/repositories and accession number(s) can be found at: NCBI, PRJNA1176302.
