# Supplementary material for: Grape seed proanthocyanidins improves growth performance, antioxidative capacity, and intestinal microbiota in growing pigs
Source: Front Microbiol. 2024 Nov 20;15:1501211. doi: 10.3389/fmicb.2024.1501211 (PMC11615056; doi:10.3389/fmicb.2024.1501211)
Supplement: Supplementary file 1 [file Table_1.DOC]

Supplementary Material

# Supplementary Tables

**Table S1** The numbers of assembled reads

| **Sample name** | **Total reads** | **Combined reads** | **Uncombined reads** | **Percent combined (%)** |
| --- | --- | --- | --- | --- |
| Con.1 | 32011 | 31222 | 1197 | 97.53522 |
| Con.2 | 38759 | 37841 | 1210 | 97.63152 |
| Con.3 | 30312 | 29676 | 994 | 97.90182 |
| Con.4 | 31442 | 30773 | 948 | 97.87227 |
| Con.5 | 31458 | 30798 | 875 | 97.90196 |
| Con.6 | 34858 | 34004 | 1274 | 97.55006 |
| GSP15.1 | 34761 | 33871 | 1227 | 97.43966 |
| GSP15.2 | 30612 | 29942 | 955 | 97.81132 |
| GSP15.3 | 34217 | 33388 | 1173 | 97.57723 |
| GSP15.4 | 33132 | 32387 | 1143 | 97.75142 |
| GSP15.5 | 37554 | 36578 | 1455 | 97.40108 |
| GSP15.6 | 34398 | 33514 | 1273 | 97.43008 |
| GSP30.1 | 32725 | 31922 | 1157 | 97.54622 |
| GSP30.2 | 36821 | 35898 | 1347 | 97.49328 |
| GSP30.3 | 32065 | 31272 | 1136 | 97.5269 |
| GSP30.4 | 32950 | 32156 | 1154 | 97.59029 |
| GSP30.5 | 37273 | 36315 | 1329 | 97.42977 |
| GSP30.6 | 34147 | 33268 | 1272 | 97.42584 |
| GSP60.1 | 39326 | 38483 | 1265 | 97.85638 |
| GSP60.2 | 37700 | 36731 | 1299 | 97.42971 |
| GSP60.3 | 35185 | 34328 | 1194 | 97.5643 |
| GSP60.4 | 31908 | 31233 | 1152 | 97.88454 |
| GSP60.5 | 39611 | 38787 | 1206 | 97.91977 |
| GSP60.6 | 34649 | 33719 | 1242 | 97.31594 |
| GSP120.1 | 33906 | 32948 | 1339 | 97.17454 |
| GSP120.2 | 35447 | 34754 | 983 | 98.04497 |
| GSP120.3 | 33160 | 32378 | 1103 | 97.64174 |
| GSP120.4 | 33377 | 32594 | 1117 | 97.65407 |
| GSP120.5 | 30967 | 30256 | 1003 | 97.70401 |
| GSP120.6 | 32278 | 31537 | 1040 | 97.70432 |
| Total | 1027009 | 1002573 | 35062 | 97.62066 |

**Table S2** The alpha diversity index of each group

| **Items** | **Dietary treatment** | | | | | **SEM** | ***P*** |
| --- | --- | --- | --- | --- | --- | --- | --- |
|  | **CON** | **15** | **30** | **60** | **120** |  |  |
| Chao1 | 628.60 | 609.26 | 664.02 | 735.53 | 555.64 | 26.74 | 0.296 |
| PD | 48.87 | 45.70 | 49.99 | 50.89 | 42.08 | 1.66 | 0.459 |
| Simpson | 0.981 | 0.977 | 0.982 | 0.981 | 0.956 | 0.01 | 0.282 |
| Shannon | 5.02^ab^ | 4.94^ab^ | 5.17^a^ | 5.23^a^ | 4.71^b^ | 0.06 | 0.036 |
| ACE | 628.86 | 606.76 | 665.41 | 735.53 | 556.60 | 26.62 | 0.289 |

Data are presented as mean (n = 6) and SEM. SEM =Standard error of mean.

^a,b^Means lacking a common uppercase superscript differ (*P* < 0.05) using Tukey test.

Con = control group; 15 = 15 mg/kg GSP group; 30 = 30 mg/kg GSP group; 60 = 60 mg/kg GSP group; 120 = 120 mg/kg GSP group.

**Table S3** **Effects of dietary GSP on relative abundance of fecal microorganisms in growing pigs at phylum level.**

| **Items** | **Dietary treatment** | | | | | **SEM** | ***P*** |
| --- | --- | --- | --- | --- | --- | --- | --- |
|  | **CON** | **15** | **30** | **60** | **120** |  |  |
| *Firmicutes*, % | 53.62^b^ | 52.78^b^ | 57.24^b^ | 52.98^b^ | 64.93^a^ | 1.34 | 0.009 |
| *Bacteroidetes*, % | 31.34^a^ | 30.64^a^ | 27.88^ab^ | 32.82^a^ | 21.77^b^ | 1.13 | 0.008 |
| *Proteobacteria*, % | 6.25 | 8.87 | 8.96 | 8.74 | 7.41 | 0.73 | 0.744 |
| *Spirochaetes*, % | 3.26 | 2.74 | 1.48 | 2.09 | 2.95 | 0.29 | 0.278 |
| *Euryarchaeota*, % | 1.60 | 1.78 | 0.89 | 0.70 | 1.37 | 0.18 | 0.260 |
| *Tenericutes*, % | 0.70 | 0.74 | 1.47 | 0.79 | 0.62 | 1.21 | 0.163 |
| *Epsilonbacteraeota*, % | 1.43^a^ | 1.35^ab^ | 0.52^bc^ | 0.70^abc^ | 0.13^c^ | 0.15 | 0.016 |
| *Actinobacteria*, % | 0.22^b^ | 0.25^b^ | 0.84^a^ | 0.30^b^ | 0.30^b^ | 0.05 | <0.001 |
| *Kiritimatiellaeota*, % | 0.38 | 0.30 | 0.27 | 0.35 | 0.25 | 0.03 | 0.624 |
| *Cyanobacteria*, % | 0.22^a^ | 0.07^b^ | 0.14^ab^ | 0.24^a^ | 0.03^b^ | 0.02 | 0.014 |
| *WPS-2*, % | 0.06^b^ | 0.21^a^ | 0.07^b^ | 0.01^b^ | 0.05^b^ | 0.02 | <0.001 |
| *Acidobacteria*, % | 0.03 | 0.03 | 0.03 | 0.05 | 0.01 | 0.01 | 0.428 |
| Others, % | 0.82 | 0.24 | 0.21 | 0.22 | 0.17 | 0.12 | 0.415 |

Data are presented as mean (n = 6) and SEM. SEM =Standard error of mean.

^a,b^Means lacking a common uppercase superscript differ (*P* < 0.05) using Tukey test.

Con = control group; 15 = 15 mg/kg GSP group; 30 = 30 mg/kg GSP group; 60 = 60 mg/kg GSP group; 120 = 120 mg/kg GSP group.

**Table S4 Effects of dietary GSP on relative abundance of fecal microorganisms in growing pigs at genus level.**

| **Items** | **Dietary treatment** | | | | | **SEM** | ***P*** |
| --- | --- | --- | --- | --- | --- | --- | --- |
|  | **CON** | **15** | **30** | **60** | **120** |  |  |
| *Lactobacillus*,% | 8.72^b^ | 9.84^b^ | 9.77^b^ | 4.85^b^ | 24.68^a^ | 1.83 | 0.002 |
| *Clostridium sensu stricto 1*,% | 8.68 | 7.29 | 6.01 | 7.80 | 8.21 | 0.54 | 0.602 |
| *Succinivibrio*,% | 5.36 | 7.89 | 7.09 | 7.64 | 6.56 | 0.70 | 0.882 |
| *Prevotellaceae NK3B31 group*,% | 6.21^a^ | 4.08^bc^ | 2.96^c^ | 5.55^ab^ | 4.04^bc^ | 0.32 | 0.003 |
| *Prevotella 9*,% | 4.24^ab^ | 5.16^a^ | 5.42^a^ | 3.44^ab^ | 1.85^c^ | 0.43 | 0.046 |
| *Ruminococcaceae UCG-005*,% | 3.85 | 3.15 | 3.62 | 3.88 | 3.42 | 0.20 | 0.789 |
| *Prevotella 1*,% | 3.10^ab^ | 3.65^a^ | 2.44^ab^ | 3.08^ab^ | 1.68^b^ | 0.25 | 0.152 |
| *Treponema 2*,% | 3.32 | 2.74 | 1.48 | 2.09 | 2.95 | 0.29 | 0.279 |
| *Ruminococcaceae UCG-002*,% | 1.80 | 2.69 | 2.53 | 2.90 | 2.47 | 0.23 | 0.667 |
| *Streptococcus*,% | 1.07^b^ | 1.09^b^ | 4.80^a^ | 1.96^b^ | 2.06^b^ | 0.37 | 0.002 |
| *Rikenellaceae RC9 gut group*,% | 2.57 | 2.21 | 1.63 | 2.18 | 2.32 | 0.16 | 0.460 |
| *Terrisporobacter*,% | 2.40 | 1.91 | 1.94 | 1.86 | 2.18 | 0.15 | 0.792 |
| *Alloprevotella*,% | 2.48^a^ | 1.79^ab^ | 1.36^ab^ | 2.32^a^ | 0.88^b^ | 0.19 | 0.023 |
| *Roseburia*,% | 2.25 | 1.62 | 1.45 | 1.45 | 1.45 | 0.16 | 0.491 |
| *Prevotella 2*,% | 1.53 | 1.57 | 1.53 | 1.84 | 0.99 | 0.13 | 0.319 |
| *Ruminococcaceae*,% | 1.03 | 1.22 | 1.81 | 1.60 | 1.27 | 0.13 | 0.353 |
| *Agathobacter*,% | 1.90^a^ | 2.04^a^ | 1.32^a^ | 1.12^ab^ | 0.32^b^ | 0.17 | 0.006 |
| *Methanobrevibacter*,% | 1.47 | 1.64 | 0.85 | 0.57 | 1.29 | 0.17 | 0.271 |
| *Megasphaera*,% | 0.84 | 1.02 | 1.43 | 1.15 | 0.70 | 0.10 | 0.163 |
| *Ruminococcus 1*,% | 1.32^a^ | 0.96^ab^ | 0.67^b^ | 1.27^a^ | 0.75^b^ | 0.08 | 0.022 |
| Others, % | 35.90^b^ | 36.44^b^ | 39.59^ab^ | 41.45^a^ | 29.92^c^ | 0.94 | <0.001 |

Data are presented as mean (n = 6) and SEM. SEM =Standard error of mean.

^a,b^Means lacking a common uppercase superscript differ (*P* < 0.05) using Tukey test.

Con = control group; 15 = 15 mg/kg GSP group; 30 = 30 mg/kg GSP group; 60 = 60 mg/kg GSP group; 120 = 120 mg/kg GSP group.
